# Using milk flow rate to investigate milk ejection in the left and right breasts during simultaneous breast expression in women

**DOI:** 10.1186/1746-4358-4-10

**Published:** 2009-10-26

**Authors:** Danielle K Prime, Donna T Geddes, Diane L Spatz, Marc Robert, Naomi J Trengove, Peter E Hartmann

**Affiliations:** 1 School of Biomedical, Biomolecular and Chemical Sciences, The University of Western Australia, M310, 35 Stirling Hwy, Crawley, Western Australia, Australia; 2 School of Nursing, University of Pennsylvania, 418 Curie Blvd, Philadelphia, Pennsylvania, USA; 3 Carag AG, Bahnhofstrasse 9, Baar, Switzerland

## Abstract

**Background:**

Milk ejection is essential for a successful lactation, however techniques to measure milk ejection in women are often complex and invasive. Recent research has demonstrated that at milk ejection, milk duct diameter increased in the breast (measured by ultrasound) at the same time as milk flow rate increased (measured using a weigh balance). This study aimed to evaluate a purpose-built continuous weigh balance (Showmilk, Medela AG) to measure changes in milk flow rate from the breast to identify milk ejections during milk expression. In addition, the Showmilk was used to determine if milk ejection occurred simultaneously in both breasts during double pumping.

**Methods:**

Increased milk flow rates during single pumping were compared to simultaneous ultrasound measurements of increased milk duct diameters in 14 mothers. In addition, increases in milk flow rate were compared between the left and right breasts of 28 mothers during double pumping for 15 minutes with two separate electric breast pumps attached to two Showmilks to record milk flow rate.

**Results:**

Increased milk flow rates were associated with increased milk duct diameters during single pumping. The mean number of milk ejections was not different between the Showmilk (4.2 ± 2.0) and ultrasound (4.5 ± 1.5) techniques. Overall, 67 milk ejections were measured and of these, 48 (72%) were identified by both techniques. The left and right breasts responded synchronously with 95.5% of the flow rate increases corresponding between the breasts. The mean number of milk ejections identified by an increase in milk flow rate during double pumping was 5.1 ± 1.7 and 5.0 ± 1.7 for the left and right breasts, respectively. In addition, mothers chose the same expression vacuum for the left (-198 ± 31 mmHg) and right (193 ± 33 mmHg) breasts.

**Conclusion:**

The Showmilk can simply and non-invasively record milk ejections by measuring increases in milk flow rate that correspond with increases in milk duct diameter. For the first time measurement of milk flow rate has been used to confirm that milk ejections occur simultaneously in the left and right breasts during double pumping. The use of the Showmilk will facilitate further research into the relationship of milk ejection and milk removal.

## Background

Even though it is well known that milk ejection is critical for successful breastfeeding and milk removal [1], scientific data regarding the relationship between milk ejection and milk removal in women is lacking. Unlike ruminants that can store up to 20% of their milk volume in the cisterns [2], the majority of milk in the human mammary gland is held in the alveolar region and requires active expulsion [3].

In response to tactile stimulation of the nipple, as well as emotional and sensory stimuli [4,5], oxytocin is released from the posterior pituitary into the maternal circulation and binds to oxytocin receptors in the mammary gland. Oxytocin-receptor binding causes the myoepithelial cells surrounding the milk-filled alveoli to contract. This contraction results in the flow of milk from the alveoli through the milk ducts to the nipple. As the milk flows, the pressure within the milk ducts increases. This pressure has been measured by ductal cannulation in both animals [6-8] and women [9-11]. As the milk duct pressure increases at milk ejection, there is a corresponding transient increase in duct diameters demonstrated by ultrasound monitoring of milk ducts [12]. Although the use of ultrasound is effective, it is technically challenging and requires a highly skilled sonographer. Measurements of plasma oxytocin levels have demonstrated that oxytocin release is pulsatile with a short half life of 2 to 3 minutes [5,13,14]. Since it is known that the milk ejection reflex can be inhibited in women by stressors [13,15], invasive methodologies such as blood sampling and duct cannulation may affect oxytocin release and the results may not be indicative of normal physiology. Furthermore, while the first milk ejection is sensed by most mothers (79%) [16], subsequent milk ejections that occur during either breastfeeding or breast expression are rarely sensed.

Recently, it has been demonstrated that transient increases in milk duct diameter, measured by ultrasound, correlate with transient increases in the rate of milk flow from the nipple, measured at five second intervals during pumping of a single breast [17]. These results initiated the development of a purpose-built continuous weight measurement device (Showmilk, Medela AG) to more accurately measure milk flow rate (8 Hz) and to increase the sensitivity of the detection of milk ejections. The aim of this study was to evaluate the Showmilk by determining both its accuracy and sensitivity in identification of milk ejections in mothers, by detecting an increase in rate of milk flow. In addition, we aimed to determine if milk ejections occurred simultaneously in the left and right breasts during double pumping.

## Methods

### Participants and ethics approval

This study has two components, the validation of the Showmilk measurement system against the ultrasound method (ultrasound and expression), and the characterisation of milk ejection in the left and right breasts of mothers during simultaneous breast expression (double pumping). Forty four mothers were recruited for the study, however six mothers did not measure their milk production and were not included. In total, 38 mothers of healthy, term infants (3 to 36 weeks of age) participated in the study. Of these 38 mothers 79% (30) were primiparous, 58% (22) of the infants were male, and all infants were predominantly fed breastmilk. One mother was exclusively expressing for her infant. Fourteen mothers participated in the ultrasound and expression component of the study and had a mean age of 32 ± 5 years (range 23 to 39 years). Twenty-eight mothers participated in the double pumping component of the study and had a mean age of 34 ± 3 years (range 28 to 43 years). Four of the mothers participated in both studies. For both the ultrasound and double pumping components of the study, mothers were asked to refrain from feeding their infants from the left breast for two hours prior to the experimental visit. The Human Research Ethics Committee of The University of Western Australia approved the study. Participants were recruited from the Western Australian branch of the Australian Breastfeeding Association and Child Health Centres, and each mother completed informed, written consent.

### Determination of breastfeeding characteristics

The milk production of the left and right breasts of each mother in both arms of the study was measured over 24-28 hours during the study period. Infant milk intake was measured in the mother's home using the test weighing method [18]. Mothers were provided with a set of electronic scales (BabyWeigh Scale, Medela AG, Switzerland; resolution 2 g, accuracy ± 0.034%), a data recording sheet, and both verbal and written instructions. For the 24 to 28 hour period, mothers weighed their baby before and after each breastfeed, from each breast. Mothers also manually expressed a small (~1.0 mL) fore- and hind-milk sample at each breastfeed into 5 mL polypropylene plastic vials. These samples were measured for fat content by the creamatocrit method [19]. The single exclusively expressing mother followed the same protocol, however, she weighed the expression bottle before and after pumping to determine the volume expressed. Milk intake was normalized to 24 hours [18] and no correction was made for infant insensible water loss. Degree of fullness and breast storage capacity was calculated from the mothers' 24 hour milk production data using the method described by Kent et al [16]. Multiplication of the degree of fullness by the breast storage capacity gave the amount of milk available in the breast before the experimental expression session. One mother in the ultrasound and expression component of the study was unable to complete the 24-hour milk production measurement. This mother was breastfeeding her infant on demand, had no concerns about her breastmilk supply, and the infant was growing appropriately for age without supplementation. In this case the data collected at multiple experimental sessions was used to create a data set to estimate degree of fullness and storage capacity.

### Ultrasound monitoring of milk ducts for milk ejection

**Figure 1:**
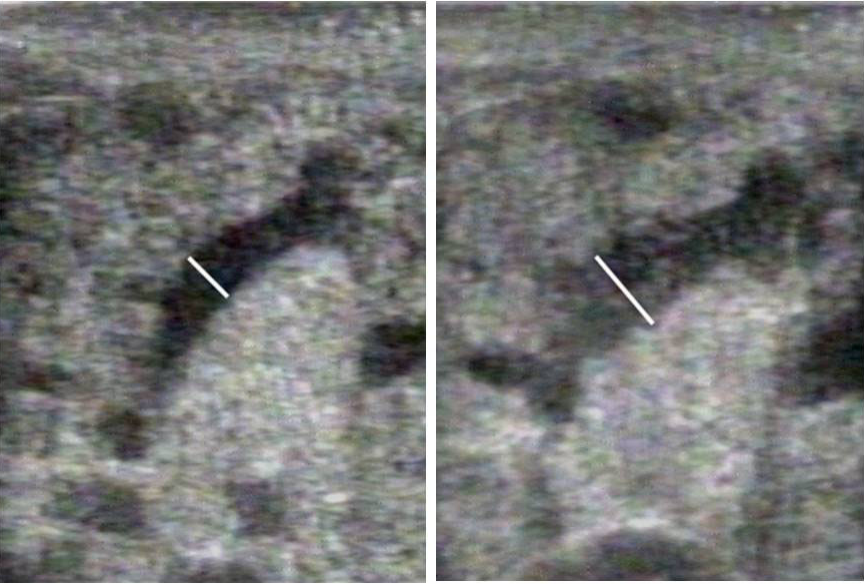
**Milk duct diameter increase, as measured by ultrasound**. These images depict a milk duct increasing in duct diameter at the time of milk ejection.

The non-expressed breast of 14 mothers was monitored with ultrasound for the duration of expression of the expressed breast to detect increases in duct diameter (milk ejections). The long axis of a main milk duct was scanned with a linear array transducer (LX7; Aplio80, Toshiba, Tokyo, Japan) at 14 MHz for high-resolution images as described previously [12]. Briefly, a milk duct was located in the lateral portion of the breast close to the base of the nipple and light pressure used to avoid either distortion or compression of the duct. The scan began when the pump was switched on and was video-taped for retrospective analysis. The diameter of the milk duct was measured every 3 to 20 seconds, that is, at times when the breast had stabilized from movement of either the mother or positioning of the transducer. Transient increases in milk duct diameter and/or milk flow within the milk duct were indicated as milk ejections according to a previously described method [12] (Figure 1 and Additional File 1).

### Milk flow rate measurement

**Figure 2:**
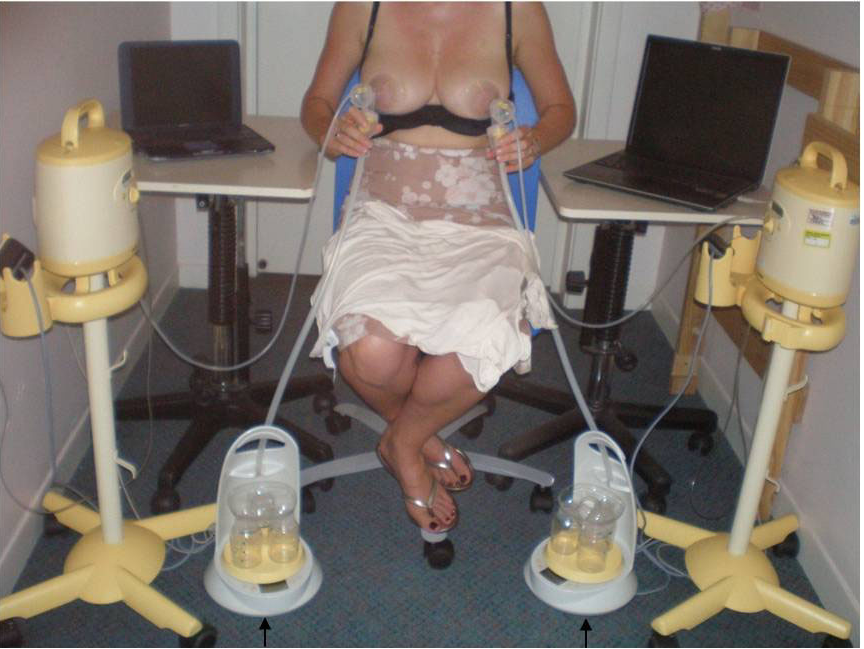
**The experimental setup for the measurement of milk flow rate in both breasts using the Showmilk technique**. The participant sat centrally and the left and right breasts were simultaneously expressed with two electric breast pumps. Milk flowed from the breast shields, through collecting tubes and onto the continuous weight measurement devices (Showmilks ↑). Vacuum was measured by two pressure transducers and data were recorded onto two notebook computers.

**Figure 3:**
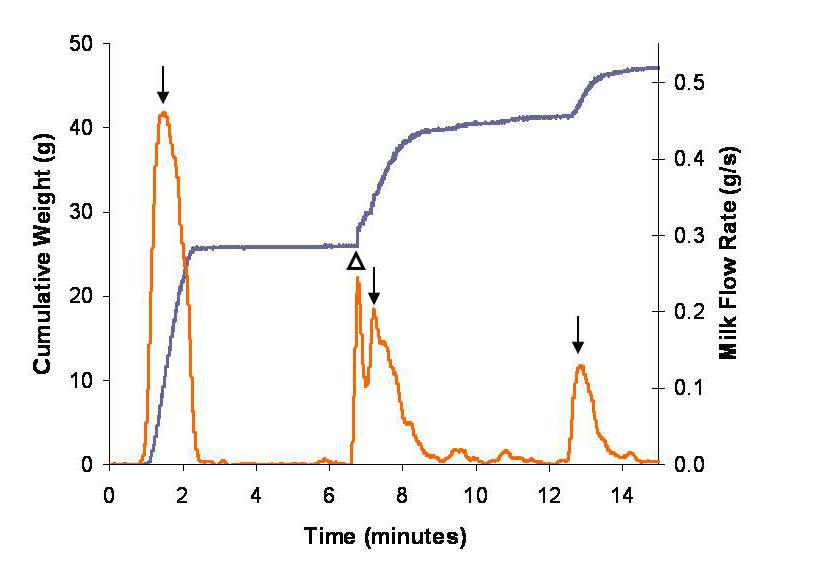
**A false peak in the milk flow rate of a mother measured by the Showmilk that was due to pooling during breast expression**. Three milk ejections (increases in milk flow rate ↓) can be observed. Milk ejections have a distinct shape with milk flow rate increasing steeply until the peak and then decreasing at a slower rate. However, in this example, after the first milk ejection, milk was pooled in the breast shield and released suddenly at the second milk ejection creating a narrow false peak (Δ) which could be mistaken for separate milk ejection. The cumulative weight of the expressed milk of the left breast is represented grey. The weight data is derived to obtain a milk flow rate value in g/s and is represented in orange.

Milk flow rate was measured using a purpose-built continuous weigh balance (Figure 2, Showmilk, Medela AG, Switzerland; resolution 0.1 g, accuracy ± 0.02% of a maximum 2 kg). Up to 24 collecting bottles can be accommodated by the Showmilk to allow collection of multiple fractions of milk during an expression session. For our studies, the Showmilk collected milk into one of three bottles placed on the weigh platform. It also has two analogue sensor inputs allowing synchronous recordings of analogue signals. It was connected to a computer using a USB interface that included interactive software (Showmilk v1.1.26, 2005 ^© ^Medela AG, Switzerland) allowing real time visualization of the flow rate data being collected. Recorded data was de-identified, saved and exported. Using a purpose-built macro (Medela AG, Switzerland), the data was then imported into a spreadsheet (Microsoft^® ^Office Excel 2003) for analysis. Cumulative weight measurements were sampled at 8 Hz, and derived to obtain the rate of milk flow (g/s, grams/second). Data readouts from the left and right breasts of a mother were overlaid, and transient increases (peaks) in milk flow rate were correlated and quantitated manually by the same rater. The milk ejection flow rate peaks have a distinct shape with a steep incline up to the peak, followed by a slower decline. This typical shape helps to differentiate a physiological flow rate increase from an artefact such as pooling of milk in the shield that is released intermittently (Figure 3).

During an expression session, the tube connecting the breast shield to the Showmilk must be positioned to allow the direct flow of milk to the weighing platform to avoid milk pooling in either the breast shield or collecting tube. The sudden movement of pooled milk is recorded as a sharp, narrow increase in milk flow rate and may be incorrectly identified as a milk ejection (Figure 3). During recording, the Showmilk is sensitive to both movement and touch, therefore the Showmilk should be positioned so that artefacts due to movement are avoided.

### Experimental protocol

Participants attended the Breast Feeding Centre of Western Australia for each study day. For the ultrasound and expression component, 14 mothers pumped the left breast for 15 minutes (after milk ejection) onto the Showmilk while the right breast was simultaneously monitored with ultrasound to detect milk duct dilations (milk ejection).

For the double pumping component, 28 mothers pumped both the left and right breasts using two separate electric breast pumps (Symphony, Medela AG, Switzerland) onto two Showmilks for 15 minutes (after milk ejection). The expressed milk travelled down collecting tubes to the Showmilks (Figure 2). During pumping the first milk ejection was identified visually as jets of milk flowing from the nipple. A small fore-milk sample (1.0 to 2.0 mL) was collected into bottle 1, then the bulk of the expressed milk was collected into bottle 2 (5.0 mL milk sample retained), and a small hind-milk sample (1.0 to 2.0 mL) was collected from bottle 3 after 14 to 15 minutes of expression. The cream content of the milk samples was measured using the creamatocrit method [19] with the Creamatocrit Plus™ centrifuge (Medela, Inc. McHenry, IL, USA). The cream values were combined with the 24 hour milk production data to determine breast fullness for each study day.

### Vacuum measurement

The strength of vacuum applied by the pump to each breast was measured in the tube that connects the pump to the shield using pressure transducers (Internal number 001-127_1, Medela AG, Switzerland) that were placed at the height of the breast shields during the pumping session. The analogue signal was recorded directly into the analogue input of the Showmilk, and automatically synchronized with the weight measurement. The vacuum for each breast was increased by the researcher until the mother indicated that she had reached her maximum comfortable vacuum [20] and the mother was blinded to the vacuum level on the breast pump displays.

### Data smoothing

As flow rate data was recorded at a very high sampling rate (8 Hz), data smoothing was required to prevent interference from the cycling vacuum of the breast pump. The customised software that was used to export data from the Showmilk included a smoothing filter (rolling average). The program also allowed manual selection of the smoothing interval (the number of time points the rolling average incorporates). In order to investigate the effect of smoothing interval on the flow rate data, milk flow rate was measured during breast expression of the left and right breasts using the Showmilk in 12 of the mothers who double pumped. Smoothing intervals between 0.5 and 15 (0.5, 0.75, 1, 1.25, 1.875, 2.5, 6.25, 10, 15) seconds were evaluated for each mother, for both the left and right breasts. The expression data for each breast was passed through the smoothing filter at each smoothing interval. The five maximum flow rate values were recorded and averaged providing a peak flow rate for each smoothing interval. This peak flow rate value is indicative of the maximum variation of the rate of milk flow.

### Statistical analysis

Milk ejections were identified from the ultrasound images while blinded to milk flow rate data. The time of duct dilation was then compared to the time of peak rate of milk flow. Comparisons of the left and right breasts, and measurement techniques (Ultrasound versus Showmilk) were performed using paired t-tests. Where the data was not normally distributed the Wilcoxon signed rank test was used (SigmaStat 3.11, 2004 ©Systat Software, Inc.). All values are presented as mean ± the standard deviation. P values less than 0.05 were considered statistically significant.

## Results

### Breastfeeding characteristics

Thirteen of the 14 mothers participating in the ultrasound and expression component completed the 24-hour milk production measurement. Their mean total milk production of 697 ± 148 g (range 428 to 921 g) was within the normal range [21]. The left and right breasts produced 350 ± 96 g (range 212 to 516 g) and 347 ± 79 g (range 191 to 455 g), respectively. There was no difference in the storage capacity of the left (160 ± 56 g) and right (171 ± 37 g) breasts for these mothers (p = 0.47). The mean milk intake of the infants at a breastfeed was 79 ± 34 g from the left and 76 ± 21 g from the right breast.

All of the mothers participating in the double pumping component completed the 24-hour milk production measurements (n = 28). Mothers had a normal milk supply [21] with a mean total milk production of 781 ± 188 g (range 467 to 1281 g). The left and right breasts produced 408 ± 129 g (range 138 to 672 g) and 373 ± 132 g (range 85 to 662), respectively and the mean storage capacity of the left and right breasts (left: 177 ± 63 g and right: 151 ± 53 g) were not significantly different (p = 0.05). The mean milk intake of the infants at a breastfeed was 64 ± 19 g from the left and 60 ± 22 g from the right breast.

### Breast expression characteristics

For the double pumping component, 28 mothers simultaneously expressed their left and right breasts with separate electric breast pumps. For each mother, there was no difference in the maximum comfortable vacuum selected for the left (-198 ± 31 mmHg) and right (-193 ± 33 mmHg) breasts (p = 0.24).

At the beginning of the expression session, the available milk was 98 ± 66 g and 90 ± 58 g for the left and right breasts, respectively. During the 15 minute expression period 62.7 ± 29.0% and 62.2 ± 27.4% of the available milk was removed from the left and right breasts, respectively. The volume of milk removed from the breasts was 62 ± 41 g from the left and 54 ± 34 g from the right breast. There were no significant differences between either the percentage of milk removed (p = 0.93) or the volume of milk removed for the left and right breasts (p = 0.59).

While there was no overall difference in the amount of milk removed from the left and right breasts, there was variation between the left and right breasts of individual mothers. The mean absolute difference of volume removed from the left and right breasts was 33 ± 29 g (range 0 to 87 g). Furthermore, the mean absolute difference of the percentage of available milk removed between the left and right breasts was 23.7 ± 20.2% (range 0 to 74%).

### Milk ejections measured by ultrasound and milk flow rate

#### Ultrasound and expression

**Figure 4:**
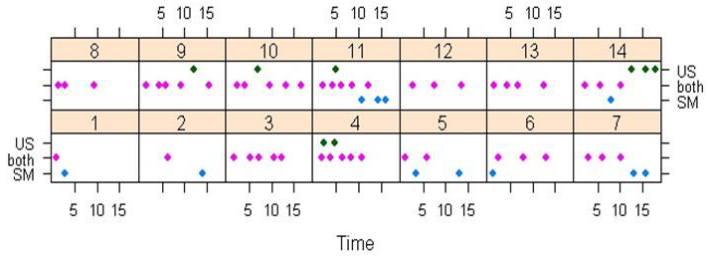
**Milk ejections detected by the ultrasound and Showmilk techniques for each mother during breast expression**. Milk ejections detected during breast expression (15 minute duration from the first milk ejection) for each mother (1-14). Milk ejections detected by both the ultrasound and Showmilk techniques are presented in pink, as the average time of detection for the two methods. Those detected only by the Showmilk (SM) are presented in blue, while those detected only by ultrasound (US) presented in green.

**Figure 5:**
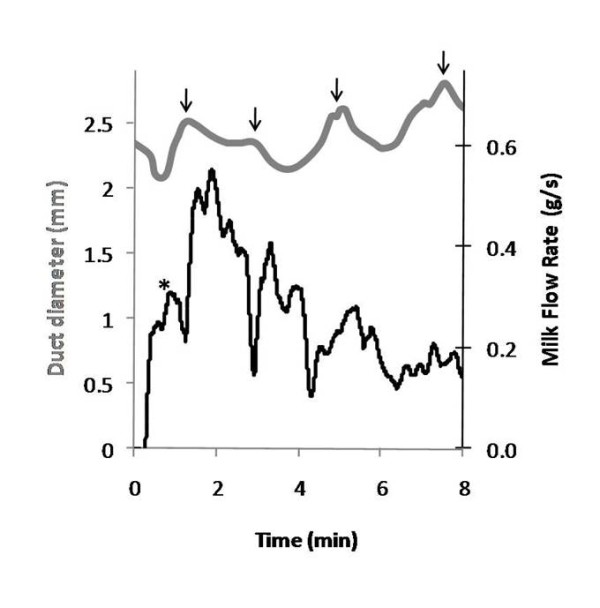
**Milk duct diameter measured with ultrasound and milk flow rate recordings by the Showmilk in a mother expressing one breast with an electric pump**. Milk duct diameter (grey) recorded by ultrasound in the right breast and milk flow rate (black) recorded in the expressed left breast of a mother by the Showmilk. Increases in milk duct diameter correspond to increases in milk flow rate (↓). A pre-milk ejection (*) can be identified as a short increase in flow rate before a greater increase in flow rate in the first minute of expression. The pre-milk ejection corresponds to a decrease in duct diameter that is most likely the end of a spontaneous milk ejection that occurred before pumping.

During the 15 minute expression session, the average number of milk ejections recorded by the two techniques was 4.2 ± 2.0 for ultrasound and 4.5 ± 1.5 for milk flow rate. Overall, 67 milk ejections were measured and of these, 48 (72%) were identified by both techniques (Figure 4). Four of the 14 mothers had all milk ejections detected by both techniques, while 8 of the remaining 10 mothers had an under or overestimation of the number of milk ejections by less than or equal to 2 (Figure 4). Changes in milk flow rate measured by the Showmilk were delayed by 42 ± 38 seconds compared to ultrasound duct diameter changes. Of the 67 milk ejections, 8 (12%) were detected by ultrasound as an increase in duct diameter without a corresponding increase in milk flow rate. Half of these (n = 4) occurred in the last 3 minutes of expression, and only one occurred in the first 4.5 minutes of expression. Furthermore, 11 (16%) of the 67 milk ejections demonstrated an increase in flow rate that did not correlate with an increase in duct diameter and 7 (64%) of these occurred in the last 5 minutes of expression. Overall, 11 of the 19 milk ejections (58%) identified by only one technique occurred in the last 5 minutes of expression. An increase in milk flow was observed prior to the first milk ejection in 5 mothers ('pre-milk ejection'; Figure 5). The peak flow rates were significantly lower (p = 0.01) for the 'pre-milk ejections' compared to the first milk ejection. The 'pre-milk ejections' occurred 20 ± 9 seconds after stimulation mode began, and had a peak flow rate of 0.19 ± 0.09 g/s. The first milk ejection (confirmed by ultrasound) occurred 111 ± 110 seconds later and corresponded to a peak flow rate of 0.49 ± 0.19 g/s.

#### Double pumping

**Figure 6:**
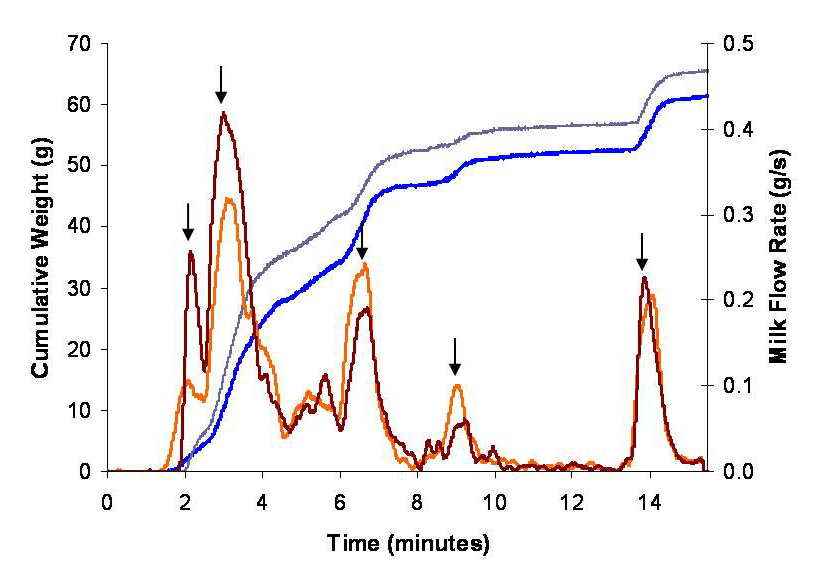
**Milk ejections in the left and right breasts of a mother measured by the milk flow rate method (Showmilk) during double pumping with an electric breast pump**. The left and right breasts during double pumping show the same timing of milk ejection. Six milk ejections in both breasts are evident as increasing flow followed by decreasing flow (↓). The cumulative weight of the expressed milk is represented as an increasing line for both the left (blue) and right (grey) breasts. The weight data was derived to obtain a milk flow rate value in g/s for the left (orange) and right (red) breasts.

**Figure 7:**
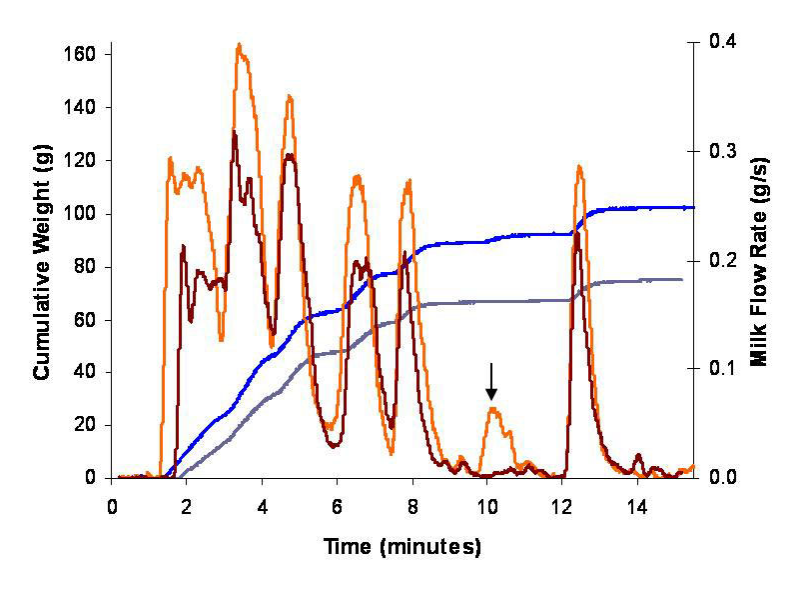
**A unilateral milk flow rate response of a mother measured by the Showmilk during double pumping**. A milk ejection was identified in the left breast at 10 minutes and 18 seconds (↓) but not in the right breast at that time. Typically these incidents occur in the final 5 minutes of breast expression. The cumulative weight of the expressed milk is represented for both the left (blue) and right (grey) breasts. The weight data was derived to obtain a milk flow rate value in g/s for the left (orange) and right (red) breasts.

**Figure 8:**
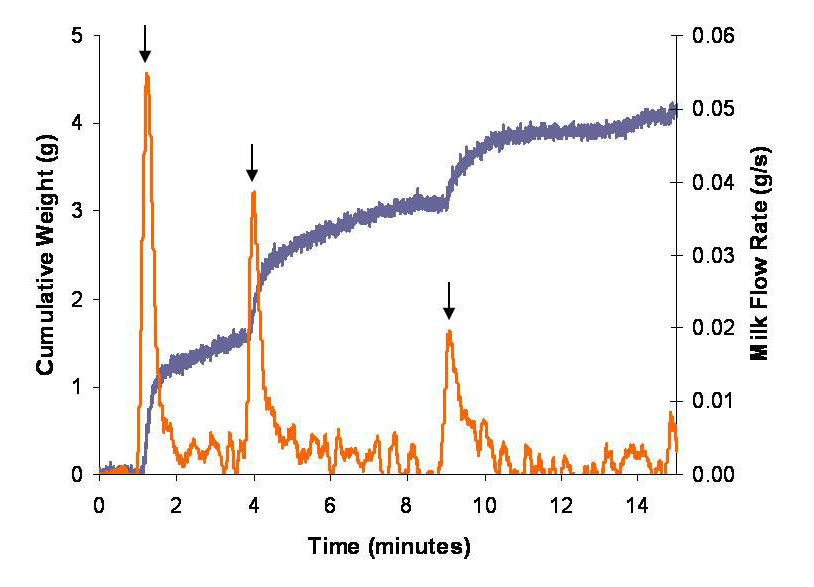
**Sensitivity of the milk flow rate method (Showmilk) in a mother with a low expression volume**. The Showmilk demonstrated three milk ejections (increases in milk flow rate, ↓). This mother only expressed 4.2 mL from the left breast. The cumulative weight of the expressed milk of the left breast is represented in grey. The weight data was derived to obtain a milk flow rate value in g/s and is represented in orange.

During the 15 minute double pumping sessions, 287 milk ejections were observed in the 28 mothers, counting left and right breasts separately, and 95.5% (n = 274) occurred simultaneously in both the left and right breasts (Figure 6). Only 3 of the 13 occasions of unilateral flow peaks (Figure 7) occurred in the first 5 minutes of expression. The mean number of milk ejections during the 15 minute expression period measured by the Showmilk was 5.1 ± 1.7 (range 3 to 8) for the left breast and 5.0 ± 1.7 (range 3 to 8) for the right breast. The highest flow rate occurred 91 ± 76 seconds and 81 ± 76 seconds after jets of milk were first observed from the nipple of the left and right breasts, respectively. The highest flow rate always occurred at either the first or second increase in milk flow, with the first increase in milk flow being the highest 62.5% of the time. The mean peak flow rate (for all milk ejections) was 0.34 ± 0.13 g/s (range 0.12 to 0.65 g/s) for the left breast and 0.33 ± 0.15 g/s (range 0.12 to 0.72 g/s) for the right breast. The Showmilk was found to be sensitive enough to record milk ejections even when a low milk volume was expressed (Figure 8).

### Data smoothing

**Figure 9:**
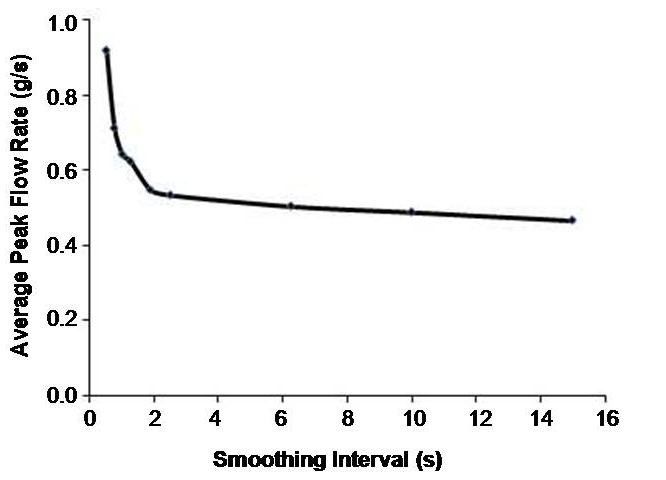
**Variation in peak milk flow rate measured by the Showmilk using different smoothing intervals**. The effect of smoothing interval on peak milk flow rate. Each point represents the average of 5 maximal peak flow rates for the left and right breasts of 12 mothers when smoothed at the appropriate interval. Erratic variation in the maximal flow rate was not observed between 2.5 and 15 seconds.

Using smoothing intervals between 0.5 and 2.5 seconds with the exported flow rate data from the Showmilk, variation in the maximal milk flow rates was observed. This variation was not apparent using smoothing intervals between 2.5 and 15 seconds (Figure 9).

## Discussion

The current study presents an accurate, sensitive and simple method for measuring increases and decreases in rate of flow of milk that represent milk ejection during expression of breastmilk with an electric pump. This method utilises a continuous weighing device with customised software (Showmilk, Medela AG) that records milk flow during breast expression. Increases and decreases in milk flow rate recorded by the Showmilk agreed 72% of the time with duct diameter changes measured by ultrasound (Figures 4 and 5). In addition, milk ejection occurred simultaneously in both breasts during double pumping.

The average number of identified milk ejections were similar between the ultrasound (4.2) and Showmilk (4.5) techniques in this study. These values are in agreement with previous research using ultrasound during breast expression for 15 minutes that found an average of 4.3 [20] and 4.4 [17] milk ejections. Furthermore, a study comparing intraductal pressure to observed milk flow during 15 minutes of breast expression [9] demonstrated 4 to 6 milk ejections. Interestingly, studies of breastfeeding using intraductal pressure measurements [10] and blood sampling for oxytocin [5] show similar numbers of milk ejections (4.4 and 3 to 5, respectively) without controlling for the length of breastfeeding. There are inherent difficulties when comparing the different techniques used to detect milk ejection, and as a result no technique has been classed as the gold standard. Each technique has measurement difficulties such as low pressures obtained by intraductal pressure measurement [22] and the influence of blood sampling rate, which can lead to under or overestimation of the number of milk ejections (oxytocin release). In addition, these invasive methodologies risk inhibiting the milk ejection reflex [15].

Compared to ultrasound, the Showmilk is cost-effective, requires little training and is portable. The technique is, however, dependent on the milk being removed from the breast, therefore the accuracy depends, in part, on both the effectiveness of the breast pump and the pumping performance of the mother. The results of this study are comparable to those obtained using other techniques to measure milk ejection, and it is concluded that the Showmilk is as accurate as the other techniques. In addition to detecting milk ejections, the Showmilk was also able to determine the time and relative strength (peak flow rate) of the milk ejections. This information could aid in the optimisation of the duration of the pumping session for individual mothers during established lactation.

The Showmilk detected all milk ejections imaged by ultrasound in 4 of the 14 women, and in 8 of the remaining 10 women the number of milk ejections were either under or over estimated by less than or equal to 2 milk ejections (Figure 4). There were 19 occasions (28%) where there was disagreement in the detection of milk ejection with ultrasound imaging and the Showmilk (Figure 4). In 8 of these 19 occasions, an increase in milk duct diameter was observed with ultrasound and no milk flow was detected. Of these, half occurred in the last 5 minutes of expression when the breast was becoming progressively drained. This phenomenon has also been observed in previous studies [17,23].

On 11 of these 19 occasions, increased milk flow rates were observed with the Showmilk, without corresponding increases in milk duct diameter and the majority of these milk flow rate increases occurred in the last 5 minutes of expression (Figure 4). Aberrant milk flow rate recordings can occur with the release of pooled milk in the breast shield tunnel, resulting in the possibility of overestimating the number of milk ejections. As decreasing amounts of milk are removed from the breast it is possible the frequency of this artefact may increase. For this study, every effort was made to minimise these occurrences. Furthermore, it is worth noting that the ultrasound monitoring of milk ducts was performed in the non-expressed breast, whereas the expressed breast was changing in the degree of breast fullness due to milk removal. It is possible that milk duct diameter changes became less obvious over time in the non-expressed breast due to milk accumulating in the main ducts. Alternatively, the monitored milk duct may have increased only slightly at milk ejection and was not detected due to the limits of resolution of ultrasound. Also, whether all milk ducts increase in diameter simultaneously at milk ejection has not yet been studied. One animal study [24] found that neighbouring lobules of the mammary gland can respond differently to oxytocin. After suckling, two neighbouring lobules were observed, one with milk-filled alveoli, and one with empty alveoli. If different areas of the breast are responding differently at milk ejection, then not all milk ducts may be experiencing milk flow at the same time. The technique of ultrasound used in this study can only monitor one or two milk ducts accurately due to their erratic course [3]. Further investigation is required to clarify the timing of ductal responses to oxytocin in women, and determine why the sensitivity of both techniques decreased after ten minutes of expression.

Pre-milk ejections were observed with the Showmilk after only 20 seconds of pumping. These milk flow rate increases had significantly lower peak flow rates and were not associated with increases in milk duct diameter, while the subsequent milk ejection was observed by both milk flow rate and duct diameter increases. Small volumes (approximately 2.5 mL) of milk removed prior to milk ejection during pumping have been reported previously [16]. This milk may represent the milk present in the milk ducts that has been drawn from the duct by vacuum of the breast pump. However, we observed milk jets during this pre-milk ejection volume, suggesting an active role of the breast (positive pressure). In one case, a decrease in milk duct diameter was observed at a time where there was a small increase in milk flow rate (Figure 5). Therefore we suspect these pre-milk ejection volumes are being expressed at the end of a milk ejection that occurred prior to the beginning of breast pumping. Unfortunately, due to the lag time between changes in duct diameter and milk flow rate recordings, the decrease in duct diameter may not have been observed for all mothers. There are two reasons why it is critical that the pump be switched from the stimulation to expression mode during these pre-ejection episodes when jets of milk are observed at the nipple. Firstly, the stimulation phase is not effective at removing milk from the breast and therefore the duration of the measured milk ejection (by flow rate) will be artificially reduced. Secondly, these episodes are shorter and have a slower flow rate and therefore can be easily recognised and accounted for when analysing the flow rate data.

The Showmilk system allowed us, for the first time, to measure simultaneous milk ejections in both breasts during double pumping. It is assumed that milk ejection occurs at the same time in both breasts due to the systemic release of oxytocin; however, no study has yet confirmed this in lactating women. Nor has there been an assessment of how both breasts respond to the same stimulation by a breast pump. We found that the left and right breasts responded with simultaneous milk ejections 95.5% of the time, confirming that indeed most of the time milk ejection occurs in both breasts at the same time. Furthermore, with regard to milk ejection, this allows us to conclude that measurement of one breast is indicative of the process in both breasts. It can now also be assumed that monitoring a milk duct with ultrasound in one breast is consistent with duct dilations (milk ejection), in the opposite breast. These conclusions allowed us to conduct the other arm of the study, looking at ultrasound and milk expression simultaneously.

During 15 minutes of double pumping we measured the same number of milk ejections (5.0) in the left and right breasts. We did, however, find the number of milk ejections to be highly variable between mothers (3-8 milk ejections). These findings are consistent with other breast expression [17,20] and breastfeeding [12] studies.

Unilateral milk flow rate increases were observed during double pumping, with the majority of discrepancies occurring toward the end of the expression session as the breast was emptied, similar to findings of the ultrasound and Showmilk component. The Showmilk will only record an increase in milk flow rate if there is sufficient milk in the breast to be removed by the pump. Therefore, if there is little milk available, it is possible milk may not flow despite milk ejection. In addition, during double pumping there may be large discrepancies between either the amount of milk in the breasts, or the emptying rates, which would also account for a milk ejection being detected in one breast and not the other. Despite this drawback, the Showmilk was still able to detect milk ejections even when low milk volumes were expressed (Figure 8), indicating that it may be possible to use this technique to detect milk ejections in mothers with low milk production.

The Showmilk records milk flow rate at a frequency of 8 Hz. Due to this high sensitivity, the recorded data requires smoothing to improve analysis. We investigated the effect of breast pump cycle frequency on milk flow rate to determine the appropriate smoothing parameters. The Symphony electric breast pump ranges from 45 to 78 vacuum cycles/minute. One vacuum cycle involves the application of vacuum to the nipple with the milk release valve closed off, followed by the release of the vacuum resulting in the opening of the milk release valve and milk flow from the breast shield, thus pulses of milk flow from the shield in 0.8-1.3 second cycles. We determined that a smoothing interval between 2.5 and 15 seconds provided a consistent measure of the rate of milk flow from the breast, therefore a smoothing interval of 10 seconds was used for this study (Figure 9).

During double pumping all electric breast pumps apply the same vacuum (set by the mother) to both breasts and do not allow independent adjustment of the applied vacuum. Because we pumped each breast independently we were able to adjust the vacuum separately according to the mothers' comfort. We found that the breast expression vacuum chosen by mothers for the left and right breasts was not different. This suggests that for healthy mothers in established lactation there is probably no advantage in regulating the vacuum levels separately for each breast during double expression. While both the vacuum and number of milk ejections did not differ between breasts, the amount of milk removed from each breast was highly variable. The percentage of available milk removed varied by almost 25% on average between the left and right breasts of a mother, despite the same vacuum being applied to the breast. Further research is required to identify factors other than milk ejection and vacuum level that may contribute to the variation in the proportion of available milk that can be removed from the breast by a breast pump.

In conclusion, the Showmilk records milk flow rate, using continuous weighing during breast expression and changes in the flow rate are indicative of milk ejection. The advantages are that it is simple, non-invasive and portable. Using this methodology we confirmed, in women, the hypothesis that milk ejection occurs synchronously in both the left and right breasts. The Showmilk is a simple technique that can be used in both clinical and research applications to undertake further investigation into milk ejection and elucidate factors influencing milk removal from the lactating breast.

## Conclusion

Measurement of milk flow rate using a custom built continuous weigh device (Showmilk) during expression is an accurate and simple method of evaluating milk ejection. This study is the first to measure milk ejection simultaneously in the left and right breasts and has confirmed that milk ejection occurs at the same time in both breasts. This is important, as it indicates that measurement of the milk ejection response in one breast is indicative of the other. In addition, no differences were found between the expression vacuum chosen for the left and right breasts. These results suggest that there is no need for breast pumps to have separate controls for the left and right breasts when used for healthy mothers without pathology. This method will enable further research into the factors influencing milk removal during breast expression.

## Competing interests

The scholarship of DKP and the salaries of DTG and NJT are partly funded by a research grant provided by Medela AG. Medela AG patent applications 'Use of a breast pump' (AT395094 - 2008/05/15; US2004122358 - 2004/06/24; US2004122357-2004/06/24) include continuous weight measurement. The authors Donna T Geddes (nee Ramsay) and Peter E Hartmann are listed as inventors on these patents.

## Authors' contributions

DKP carried out the experiments described, analysed the data and drafted the manuscript. DTG carried out the ultrasonography component of the study, and edited the manuscript. DLS participated in the study design and experimentation of the expression and ultrasound component, as well as editing the manuscript. MR was responsible for the design and ongoing engineering aspects of the Showmilk and its related components. NJT was involved in study design and editing of the manuscript. PEH participated in the design and coordination, as well as helping to draft the manuscript. All authors read and approved the final manuscript.

## Supplementary Material

Additional file 1 **Increase in milk duct visualised by ultrasound at milk ejection**. This movie depicts milk duct dilation in the right breast at milk ejection when monitored with ultrasound. The left breast was expressed with an electric breast pump. As milk duct diameter increases milk flow can be visualised as echogenic (white flecks) moving toward the nipple (right side of the image). (The file is MPG format and can be viewed with either Apple QuickTime Player or Microsoft Windows Media Player). Click here for file
